# The Influence of Extreme Heat on Police and Fire Department Services in 23 U.S. Cities

**DOI:** 10.1029/2020GH000282

**Published:** 2020-11-06

**Authors:** Augusta Williams, Larissa McDonogh‐Wong, John D. Spengler

**Affiliations:** ^1^ Department of Environmental Health Harvard T.H. Chan School of Public Health Boston MA USA; ^2^ Center for Climate, Health, and the Global Environment Harvard T.H. Chan School of Public Health Boston MA USA

**Keywords:** extreme heat, emergency services, public health

## Abstract

Recent research suggests that extreme heat affects the demand for emergency services, including police and fire department incidents. Yet there is limited understanding of impacts across U.S. cities, with varying population sizes, and between different climates. This study sought to examine the daily utilization of police and fire department services, during hot days in 23 U.S. cities representing six climate zones using relative risk (RR) and time series analyses of daily police and fire department incidents. The warm season analyses utilized three temperature metrics: daily maximum temperature (T_MAX_), daily maximum heat index (HI_MAX_), and the preceding daily minimum temperature (T_MIN_). Across these cities, the RR of police department incidents on days where T_MAX_ was at or above the 95th percentile significantly increased within a range from 3% (95% confidence interval [CI]: 0.3%, 6.3%) to 57% (95% CI: 24.5%, 89.7%), compared with a nonhot day. At the same temperature thresholds, the RR of fire department dispatches increased from 6% (95% CI: 3.0%, 8.6%) to 18% (95% CI: 15.2%, 21.6%). These results remained consistent across temperature metrics and consecutive days of extreme heat. The estimated effects of daily maximum temperature, daily maximum heat index, and daily minimum temperature were nonlinear for police and fire department incidents across all cities. These findings inform climate change adaptation strategies, preparing budgets and personnel for emergency agencies to ensure resilience as periods of extreme heat increase in frequency, severity, and duration.

## Key Points

- Forty‐one percent (79%) of cities had higher relative risk of police (fire) department dispatches on days when the T_MAX_> = 95th percentile
- The estimated effects of daily T_MAX_, daily HI_MAX_, and daily T_MIN_ were nonlinear for police and fire department incidents across all cities
- These findings can inform climate change adaptation strategies to enhance the resilience of budgets and first responders in a hotter world

## 1 Introduction

Global climate change is increasing the frequency, duration, and severity of extreme heat events. By 2050 estimates project that there could be an increase of 20–30 more of hot days at or above 90°F annually (Vose et al., 2017). Extreme heat has been found to significantly impact human health, inducing negative health symptoms, exacerbating many preexisting conditions, and even resulting in death (Basu, 2009; Reidmiller et al., 2018; U.S. Global Change Research Program, 2014), making it of paramount public health concern.

Previous literature has also found that extreme heat is associated with changed cognitive and neurologic function, as well as behavior. During extreme heat events, there can be decrements in cognitive function (Cedeño Laurent et al., 2018), inhibited sleep quality and duration (Cedeño Laurent et al., 2018; Williams et al., 2019), and increases in impulsive behavior (Gockel et al., 2014). Extreme heat has also been found to result in increased aggression (Anderson, 2001; Butke & Sheridan, 2010) and violent behavior (Anderson, 2001; Michel et al., 2016; Rotton & Cohn, 2000, 2004; Tiihonen et al., 2017). Projections within the United States have demonstrated that the United States may experience 2.3–3.2 million more violent crimes by the end of the century depending on greenhouse gas emissions trajectories (Harp & Karnauskas, 2020).

Recent findings have hypothesized that these pathways can result in impacts to societal services. An analysis of police‐initiated traffic stops in the United States found that the number of police stops peaks around 29°C, declining with temperature below or above that threshold, even though violent crime is higher at higher temperatures (Obradovich et al., 2018). However, this captures police‐initiated incidents, not necessarily the overall need for police services by the public. In Boston, MA, our recent paper found a 2% increase in local police dispatches, a 10% increase in local fire dispatches, and a 9% increase in local emergency medical service dispatches on warm‐season days that had a daily maximum temperature of at least 90°F (Williams et al., 2020). Nationally, only 4% of fire department responses are for fires, and a large majority, nearly 65%, are for medical response calls (US FEMA, 2018). There is a large body of scientific work on the impact of heat on ambulance calls (Bassil et al., 2011; Cheng, Xu, Zhao, Xie, Yang, et al., 2016; Cheng, Xu, Zhao, Xie, Zhang, et al., 2016; Guo, 2017; Papadakis et al., 2018) but not explicitly the burden of medical calls that falls to local fire departments. There is limited research on these heat impacts on police and fire departments across the United States, by various city sizes, and between different climates.

Despite the growing amount of evidence on the impacts of extreme heat on emergency services, climate action plans rarely mention heat preparedness for emergency services apart from preparedness for medical professionals and healthcare systems. It is critical that local, state, and federal governments consider not only the impact of heat on socially or medically vulnerable populations but also how those incidents will affect the larger system response. As the impacts of extreme heat events continue to escalate, cities and other government entities must think critically about how to prepare for these challenges in emergency service agency's budgets, personnel, and operations. This study seeks to expand upon the research of previous work around extreme heat and emergency services by examining the utilization of emergency services, specifically police and fire department services, during hot days in 23 cities across six climate zones across the United States.

## 2 Materials and Methods

### 2.1 Police and Fire Incident Data

Cities were chosen to cover a variety of regions, climate zones, and population sizes. Counts for daily fire and police department service were accessed in two ways: either through download from public databases or through direct outreach to city agencies. Data requests to local dispatch officials were uniform, inquiring for agency‐specific incident counts. Emergency service dispatch systems are locally determined, so some cities only record police or fire incidents or calls as compared to all dispatches or specific incident types (e.g., crime) as there is a lack of standardized metrics for emergency response data (Neusteter et al., 2019). Therefore, the type of data available for each city differs slightly (Table 1). The majority of cities reported calls or incidents that police or fire departments were dispatched to, while some reported crime dispatches only for police, which is only a subset of the types of calls police receive. Two cities reported fire and emergency medical service dispatches, where these services are combined within one agency. Throughout the manuscript, the terms dispatches and response refer to all call types accessed. Across these data sets, there was an average of 2,795.7 days (7.7 years) of police department data across 17 cities and 2,954.4 days (8.1 years) of fire department data for 14 cities. There were eight cities that had both police and fire department data available.

**Table 1:** Overview and Descriptive Statistics of Police and Fire Department Data and Meteorological Conditions for U.S. Cities of Interest

Agency	City	2018 population	Data available	Time period available	Full year					Warm season restricted				
					T_MEAN_ (°F)	T_MIN_ (°F)	T_MAX_ (°F)	Total calls	Daily mean number of calls	T_MEAN_ (°F)	T_MIN_ (°F)	T_MAX_ (°F)	Total calls	Daily mean number of calls
Police	Atlanta, GA	498,073	Incidents	01/2015–12/2018	62.74	53.65	72.98	4,085,732	2,796.5	76.03	67.45	86.48	1,686,337	2,755.5
	Austin, TX	962,243	Crime	01/2015–12/2018	69.51	52.15	71.77	148,287	101.5	81.18	73.05	92.58	62,347	101.9
	Baltimore, MD	602,495	Crime	01/2012–09/2018	55.98	36.89	53.36	324,494	133.3	71.75	64.84	81.59	148,430	142.4
	Cambridge, MA	118,967	Crime	01/2009–03/2018	51.83	45.05	58.89	66,347	19.6	67.83	60.99	75.36	29,812	21.6
	Chicago, IL	2,722,586	Crime	03/2005–05/2006	53.10	45.73	60.32	267,521	366.5	70.59	62.58	78.42	133,132	435.1
	Detroit, MI	672,681	Crime	09/2016–04/2018	50.64	43.03	58.15	559,000	683.4	68.88	60.30	77.33	239,256	754.8
	Durham, NC	274,497	Crime	01/2014–10/2018	61.56	52.21	71.88	124,924	71.0	75.25	66.68	85.32	55,943	73.1
	Hartford, CT	124,390	Incidents	01/2005–10/2018	52.07	42.49	61.87	190,809	109.1	68.82	58.66	79.69	86,565	113.2
	Los Angeles, CA	3,949,776	Incidents	01/2010–12/2018	64.88	53.50	71.76	8,645,258	2,650.3	69.79	63.37	79.52	3,818,225	2,772.9
	Minneapolis, MN	411,452	Incidents	03/2007–09/2019	47.83	39.63	55.86	5,274,321	1,166.1	68.45	59.76	77.28	2,467,773	1,254.0
	New Orleans, LA	388,182	Incidents	01/2011–09/2019	71.38	57.16	7.20	3,995,707	1,260.9	82.14	77.07	88.31	1,745,312	1,289.0
	New York, NY	8,560,072	Incidents	01/2013–12/2018	55.39	48.60	62.63	5,756,130	1,212.3	70.78	63.72	78.79	2,535,521	1,274.8
	Philadelphia, PA	1,569,657	Crime	01/2006–09/2019	57.01	49.34	65.17	2,657,507	529.9	72.96	65.23	81.73	1,210,789	566.6
	San Diego, CA	1,390,966	Incidents	01/2014–09/2018	65.63	60.48	72.01	3,641,460	1,751.5	69.57	65.89	74.76	1,630,321	1,801.5
	San Francisco, CA	833,305	Incidents	01/2003–12/2014	58.00	—	—	1,912,916	392.8	60.66	—	—	790,090	394.5
	Seattle, WA	688,245	Crime	01/2008–10/2018	52.78	46.51	60.27	497,750	125.9	62.27	54.39	71.89	216,576	128.7
	Tempe, AZ	178,339	Crime	11/2012–10/2018	77.01	65.92	87.68	163,327	53.4	91.20	79.90	101.52	68,992	52.8
Fire	Cambridge, MA	118,967	Incidents	01/2015–12/2018	52.41	45.53	59.58	18,585	12.7	68.44	61.50	76.17	7,820	12.8
	Cary, NC	80,793	Incidents	01/2013–10/2018	61.16	51.88	71.35	49,615	23.4	74.81	66.32	84.78	21,508	23.4
	Detroit, MI	672,681	Incidents	01/2017–09/2019	50.56	42.77	58.26	57,247	60.5	68.71	59.92	77.31	26,446	64.8
	Duluth, MN	85,884	Incidents	01/2007–08/2019	40.79	32.17	49.21	909,552	196.9	60.62	51.28	70.43	419,834	215.0
	Fargo, ND	118,099	Incidents	01/2015–08/2019	44.41	34.44	53.86	49,541	29.0	65.94	54.77	76.69	22,257	30.0
	Milwaukee, WI	599,086	Incidents	01/2009–08/2019	48.97	41.58	56.27	858,985	220.5	66.60	58.76	74.71	386,323	233.7
	Minneapolis, MN	411,452	Incidents	03/2007–09/2019	47.80	39.59	55.83	514,239	113.0	68.45	59.76	77.28	228,986	116.4
	New York, NY	8,560,072	Incidents	01/2006–12/2018	55.58	49.24	62.45	3,375,198	1,540.5	71.52	65.05	79.09	1,445,216	1,574.3
Fire	San Antonio, TX	1,461,623	Incidents	01/2014–09/2019	69.41	60.73	79.90	1,969,617	981.4	80.83	73.03	91.39	819,559	992.2
	San Diego, CA	1,390,966	Incidents	01/2007–09/2019	64.14	59.06	70.32	1,695,578	364.2	68.31	64.71	73.34	734,588	369.3
	San Francisco, CA	833,305	Incidents	04/2000–10/2018	58.09	—	—	3,701,499	735.2	60.63	—	—	1,566,209	731.2
	Seattle, WA	688,245	Incidents	06/2010–10/2018	53.38	47.07	60.91	762,944	258.4	62.83	54.87	72.56	346,709	269.4
	Tempe, AZ	178,339	FEMS	04/2016–08/2019	78.17	67.15	88.79	45,115	45.1	91.11	79.69	101.51	20,398	44.4
	Washington, DC	702,455	FEMS	01/2008–07/2019	59.26	51.78	67.39	2,262,961	535.2	75.34	67.79	83.70	998,887	562.8

*Note*. T_MEAN_ = daily mean temperature, T_MIN_ = daily minimum temperature, T_MAX_ = daily maximum temperature, FEMS = fire and emergency medical services.

### 2.2 Meteorological Data

Daily maximum temperature (T_MAX_), daily minimum temperature (T_MIN_), and daily maximum heat index (HI_MAX_) were all obtained from the National Center for Environmental Information (NOAA, NCEI, 2018), using the closest airport weather station for each city. The 95th, 97th, and 99th percentiles of T_MAX_, T_MIN_, and HI_MAX_ were used to define extremes days based on the local climate for the time frame of data available for each city. The values from the preceding overnight period were used to define T_MIN_. For San Francisco, CA, the daily mean temperature (T_MEAN_) was the only temperature metric available and was used instead of T_MAX_, T_MIN_, or HI_MAX_. Single days of temperature extremes were analyzed, as well as two consecutive days at or above these thresholds.

### 2.3 Statistical Analyses

Relative risk (RR) analyses determined how fire or police dispatch changed on extreme heat days compared with nonextreme heat days. Binary variables for extreme heat day were based on whether or not the local T_MAX_, HI_MAX_, and T_MIN_ on the preceding day exceeded the 95th, 97th, or 99th percentiles of these values. RR analyses were conducted using generalized additive models with a quasi‐Poisson distribution. Time series (TS) analyses were used to assess the relationship between extreme heat thresholds (T_MAX_, T_MIN_ on the preceding day, and HI_MAX_) on agency dispatches per unit temperature (or HI). The restricted TS models used a nonparametric spline with 1 degree of freedom (df) per year to account for any long‐term trends in police or fire department services and a natural cubic spline with 2 df for T_MAX_, T_MIN_, or HI_MAX_, all determined a priori based on previous literature and to optimize the Generalized Cross Validation criteria. Sensitivity analyses on the df for T_MAX_ or HI_MAX_ did not influence the main results (not shown). In addition to long‐term trends, analyses were also controlled for day of week, referenced to Friday. All RR and TS analyses were restricted to the warm season (May–September) to reduce any seasonal confounding between extreme heat and police or fire department services.

## 3 Results

**Figure 1:**
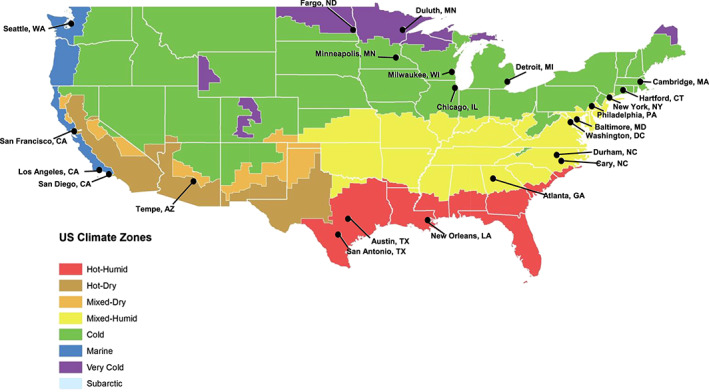
Map of the United States with selected cities identified. Color shading indicates U.S. Department of Energy Climate Zones, as adapted from the U.S. Energy Information Administration (US EIA, 2018).

A total of 37,755 warm season days was included in this study period from 2000 through 2019, across 23 U.S. cities comprising nearly 27 million people (based on 2018 U.S. Census population estimates) and were in diverse climate zones (Figure 1). Dispatches on these warm season days amounted to 16,804,911 total police department calls and 7,043,034 total fire department calls. There was an average of 819.5 police department and 374.3 fire department dispatches per day in the warm season (Table 1).

### 3.1 RR Analyses

The RR of police department incidents on a hot day with extremely high temperatures, as determined by a binary indicator in which T_MAX_ was at or above the 95th percentile, compared with a nonhot day, increased from 3–57% in 7 of the 17 cities with police data (Table 2). Increases were moderate yet still statistically significant, for Los Angeles, CA (RR = 1.03; 95% confidence interval [CI]: 1.003, 1.063), and San Diego, CA (RR = 1.04; 95% CI: 1.014, 1.056). The RR of police department incidents on these days was greatest in Chicago, IL, which had an RR = 1.57 (95% CI: 1.245, 1.897). The relative increase remained significant even with more severe temperature thresholds at the 97th and/or the 99th percentile of T_MAX_ for Atlanta, GA, Cambridge, MA, Chicago, IL, Detroit, MI, and San Diego, CA, as well as San Francisco, CA, which used T_MEAN_ instead of T_MAX_.

**Table 2:** The Relative Risk (RR) of Police and Fire Department Calls Meeting Specific Daily Maximum Temperature (T_MAX_) Thresholds Compared to All Other Days, Calculated During the Warm Season

Agency	City	T_MAX_ ≥ 95th percentile		T_MAX_ ≥ 97th percentile		T_MAX_ ≥ 99th percentile	
		RR	95% CI	RR	95% CI	RR	95% CI
Police	Atlanta, GA	**1.08**	**(1.036, 1.125)**	**1.06**	**(1.008, 1.119)**	**1.09**	**(1.000, 1.183)**
	Austin, TX	0.97	(0.908, 1.031)	0.99	(0.905, 1.070)	0.93	(0.798, 1.058)
	Baltimore, MD	1.01	(0.977, 1.035)	1.02	(0.977, 1.059)	1.01	(0.930, 1.089)
	Cambridge, MA	**1.09**	**(1.028, 1.147)**	**1.08**	**(1.009, 1.149)**	1.09	(0.971, 1.202)
	Chicago, IL	**1.57**	**(1.245, 1.897)**	**1.58**	**(1.135, 2.026)**	1.62	(0.940, 2.304)
	Detroit, MI	**1.11**	**(1.003, 1.224)**	1.11	(0.980, 1.234)	**1.22**	**(1.013, 1.426)**
	Durham, NC	1.03	(0.971, 1.087)	1.02	(0.946, 1.094)	0.94	(0.845, 1.042)
	Hartford, CT	0.97	(0.894, 1.047)	1.00	(0.909, 1.095)	0.94	(0.783, 1.103)
	Los Angeles, CA	**1.03**	**(1.003, 1.063)**	1.03	(0.990, 1.061)	1.01	(0.946, 1.068)
	Minneapolis, MN	0.99	(0.966, 1.012)	0.98	(0.956, 1.011)	0.95	(0.906, 0.994)
	New Orleans, LA	1.02	(0.998, 1.042)	**1.04**	**(1.009, 1.065)**	**1.06**	**(1.016, 1.106)**
	New York, NY	1.00	(0.978, 1.013)	1.00	(0.977, 1.022)	1.01	(0.972, 1.041)
	Philadelphia, PA	0.98	(0.956, 1.013)	1.00	(0.964, 1.034)	1.03	(0.977, 1.079)
	San Diego, CA	**1.04**	**(1.014, 1.056)**	**1.04**	**(1.013, 1.067)**	**1.07**	**(1.026, 1.112)**
	San Francisco, CA	**1.03**	**(1.006, 1.055)**	**1.04**	**(1.011, 1.073)**	**1.05**	**(1.004, 1.096)**
	Seattle, WA	0.99	(0.951, 1.020)	0.97	(0.930, 1.018)	0.95	(0.883, 1.026)
	Tempe, AZ	0.89	(0.757, 1.022)	0.98	(0.825, 1.143)	1.18	(0.886, 1.465)
Fire	Cambridge, MA	1.07	(0.958, 1.186)	**1.17**	**(1.014, 1.318)**	**1.49**	**(1.279, 1.698)**
	Cary, NC	**1.13**	**(1.066, 1.190)**	**1.16**	**(1.086, 1.242)**	**1.25**	**(1.126, 1.371)**
	Detroit, MI	**1.14**	**(1.025, 1.250)**	**1.15**	**(1.023, 1.276)**	**1.25**	**(1.034, 1.473)**
	Duluth, MN	**1.11**	**(1.079, 1.143)**	**1.13**	**(1.085, 1.170)**	**1.16**	**(1.093, 1.231)**
	Fargo, ND	**1.11**	**(1.043, 1.167)**	**1.11**	**(1.033, 1.186)**	1.05	(0.938, 1.170)
	Milwaukee, WI	**1.15**	**(1.113, 1.185)**	**1.16**	**(1.113, 1.207)**	**1.15**	**(1.083, 1.217)**
	Minneapolis, MN	**1.18**	**(1.152, 1.216)**	**1.21**	**(1.172, 1.248)**	**1.27**	**(1.212, 1.329)**
	New York, NY	**1.16**	**(1.129, 1.187)**	**1.16**	**(1.123, 1.191)**	**1.16**	**(1.099, 1.231)**
	San Antonio, TX	1.02	(1.000, 1.048)	1.01	(0.972, 1.042)	1.03	(0.966, 1.099)
	San Diego, CA	**1.16**	**(1.132, 1.188)**	**1.16**	**(1.129, 1.195)**	**1.20**	**(1.140, 1.263)**
	San Francisco, CA	**1.17**	**(1.140, 1.193)**	**1.23**	**(1.193, 1.270)**	**1.34**	**(1.283, 1.402)**
	Seattle, WA	**1.12**	**(1.065, 1.172)**	**1.12**	**(1.059, 1.189)**	1.10	(0.992, 1.216)
	Tempe, AZ	1.03	(0.950, 1.103)	1.07	(0.976, 1.163)	1.05	(0.896, 1.194)
	Washington, DC	**1.06**	**(1.030, 1.086)**	**1.05**	**(1.006, 1.087)**	**1.08**	**(1.014, 1.145)**

*Note*. Instead of hourly temperature observations, San Francisco only had daily mean temperature available for this study period so was calculated using the 95th, 97th, and 99th percentiles of daily mean temperature. Bold values indicate RRs that are significant at *p* < 0.05.

The RR for fire department incidents on hot days on days where T_MAX_ exceeded the 95th percentile significantly increased in 11 of the 14 cities with available data (Table 2). The RRs ranged from 6–18%. The greatest RRs on these days were seen in San Diego, CA (RR = 1.16; 95% CI: 1.132, 1.188), New York, NY (RR = 1.16; 95% CI: 1.129, 1.187), and Minneapolis, MN (RR = 1.18; 95% CI: 1.152, 1.216). The significance of these estimates largely remained consistent even under higher percentile values of T_MAX_, while the magnitude of the RR increased 8–49% on days when T_MAX_ exceeded the 99th percentile.

When considering alternative heat metrics, like HI_MAX_, the results remained largely unchanged, with 6 of the 17 cities with police data and 11 of the 14 cities with fire data experiencing relative increases in police and fire department incidents when the HI_MAX_ exceeded the 95th and 97th percentiles. At or above the 99th percentile of HI_MAX_, there was an increase in the RR of fire department incidents. However, New Orleans, LA, was the only city with police incident data that saw a significant increase the RR of incidents at this threshold (RR = 1.05; 95% CI: 1.007, 1.093) (supporting information Table S1). Under all percentile thresholds of T_MIN_ on the preceding day, the RR of fire department incidents continued to remain significant for the majority of cities. However, many fewer cities (3 of 17) had significant increases in the RR of police incidents using this extreme heat metric as the exposure (Table S1). The number of days meeting the specified thresholds for each city is available in Table S2, with those cities with longer durations of data availability contributing more days to their respective analyses. All RR trends remained largely consistent even when considering two consecutive days at or above the given T_MAX_, HI_MAX_, or T_MIN_ threshold (Table S3).

### 3.2 TS Analyses

#### 3.2.1 Police Department Incidents

**Figure 2:**
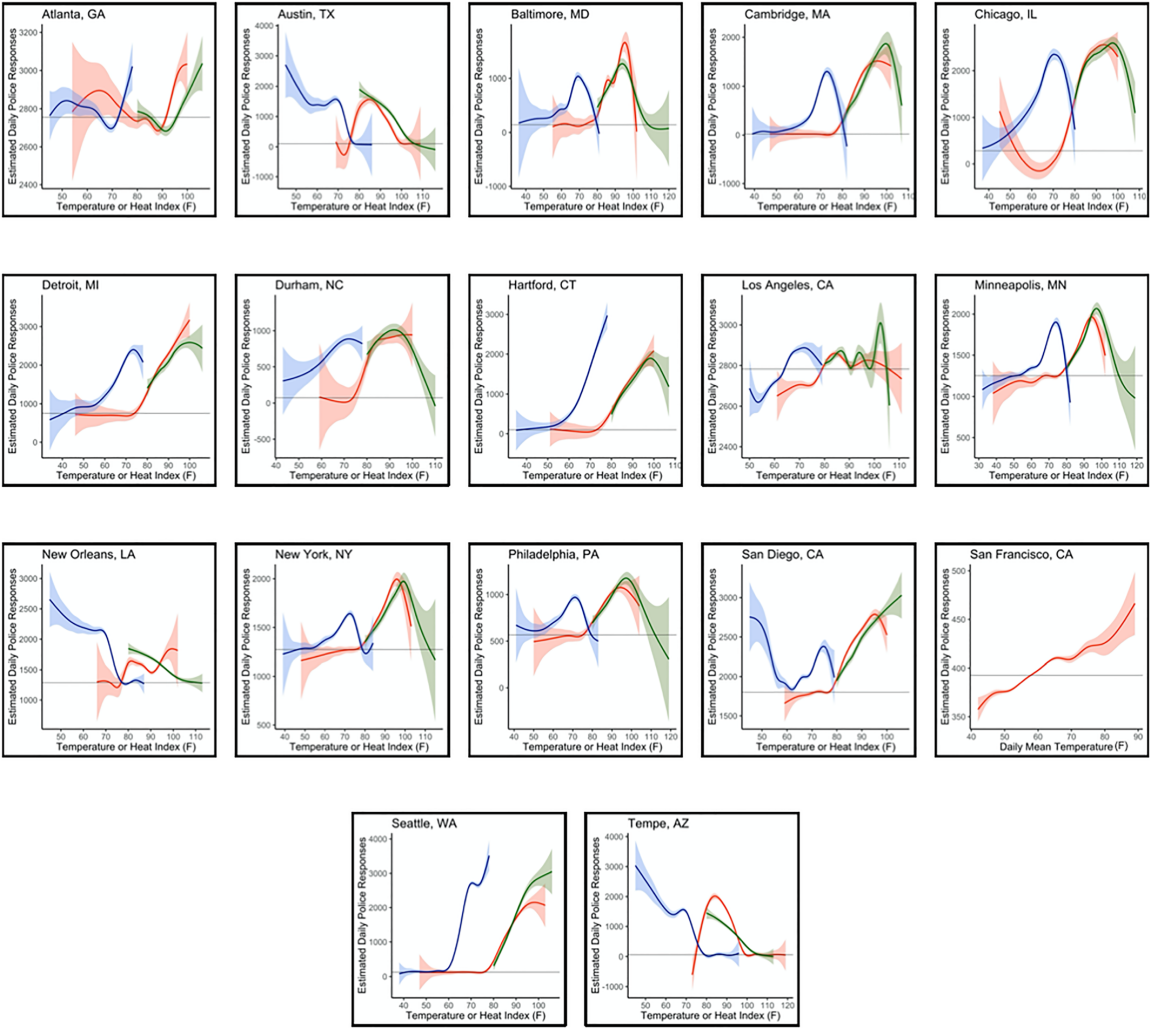
Estimated number of police calls or incidents per unit temperature (°F) or heat index (°F) during the warm season, where red indicates daily maximum temperature, green indicates daily maximum heat index, and blue indicates daily minimum temperature during the preceding day, the mean number of police calls/incidents for each city is shown by in black, and 95% confidence intervals are shaded around each respective line.

The estimated effects of T_MAX_, HI_MAX_, and T_MIN_ were nonlinear for police department incidents across all cities (Figure 2). There were few apparent patterns in police incidents based on the type of data available (e.g., incidents vs. calls) or by U.S. census region (e.g., Northeast, South, Midwest, and West). With the exception of Los Angeles, CA, all of the cities that had populations greater than one million (New York, NY, San Diego, CA, Philadelphia, PA, and Chicago, IL) had a high level of similarity in their respective patterns when assessing exposure with either T_MAX_ or HI_MAX_. Los Angeles, CA, New Orleans, LA, and Tempe, AZ, all had very different patterns for each of the chosen temperature metrics, while all other cities had some similarities across temperature metrics.

Trends also emerged based on climate zones (Figure 1). For those cities in the *Cold* climate zone (Chicago, IL, Minneapolis, MN, Cambridge, MA, Detroit, MI, and Hartford, CT), the estimated number of daily police calls increased under both high T_MAX_ and HI_MAX,_ peaking from 90–100°F. In this climate zone, the peak in daily calls was of a similar magnitude, if not larger, under the preceding day's T_MIN_. Police calls peaked around a T_MIN_ of 70°F, decreasing below that threshold, which may signal increases in police responses during days preceded by warm nights, as occurs during prolonged heat events. A similar trend was seen in the *Mixed‐Humid* climate zone (Philadelphia, PA, Durham, NC, and New York, NY) but with peaks at T_MAX_ and HI_MAX_ values closer to 100°F.

For cities in the *Marine* climate zone (Seattle, WA, and Los Angeles, CA), the estimated number of police incidents per day, on average, was greater when considering HI_MAX_ than T_MAX_ (Figure 2). In cities that were classified as either *Hot‐Humid* (Austin, TX, and New Orleans, LA) or *Hot‐Dry* (San Diego, CA, and Tempe, AZ), the preceding day's T_MIN_ resulted in the greatest number of estimated average daily police incidents, increasing as minimum temperatures decreased. This was the opposite pattern than for all other cities when analyzing the preceding day's minimum temperature.

During the warm season, a 10°F increase in T_MAX_—from 80°F to 90°F—resulted in 1.034 and 1.031 times the expected number of daily police calls in Cambridge, MA, and Hartford, CT, respectively, on average, after adjustment for the other predictors in the model. For a 10°F increase in the preceding day's T_MIN_ from 60°F to 70°F resulted in 1.030 and 1.023 times the expected number of police calls in Cambridge, MA, and Chicago, IL, respectively, and after controlling for other covariates.

#### 3.2.2 Fire Department Incidents

**Figure 3:**
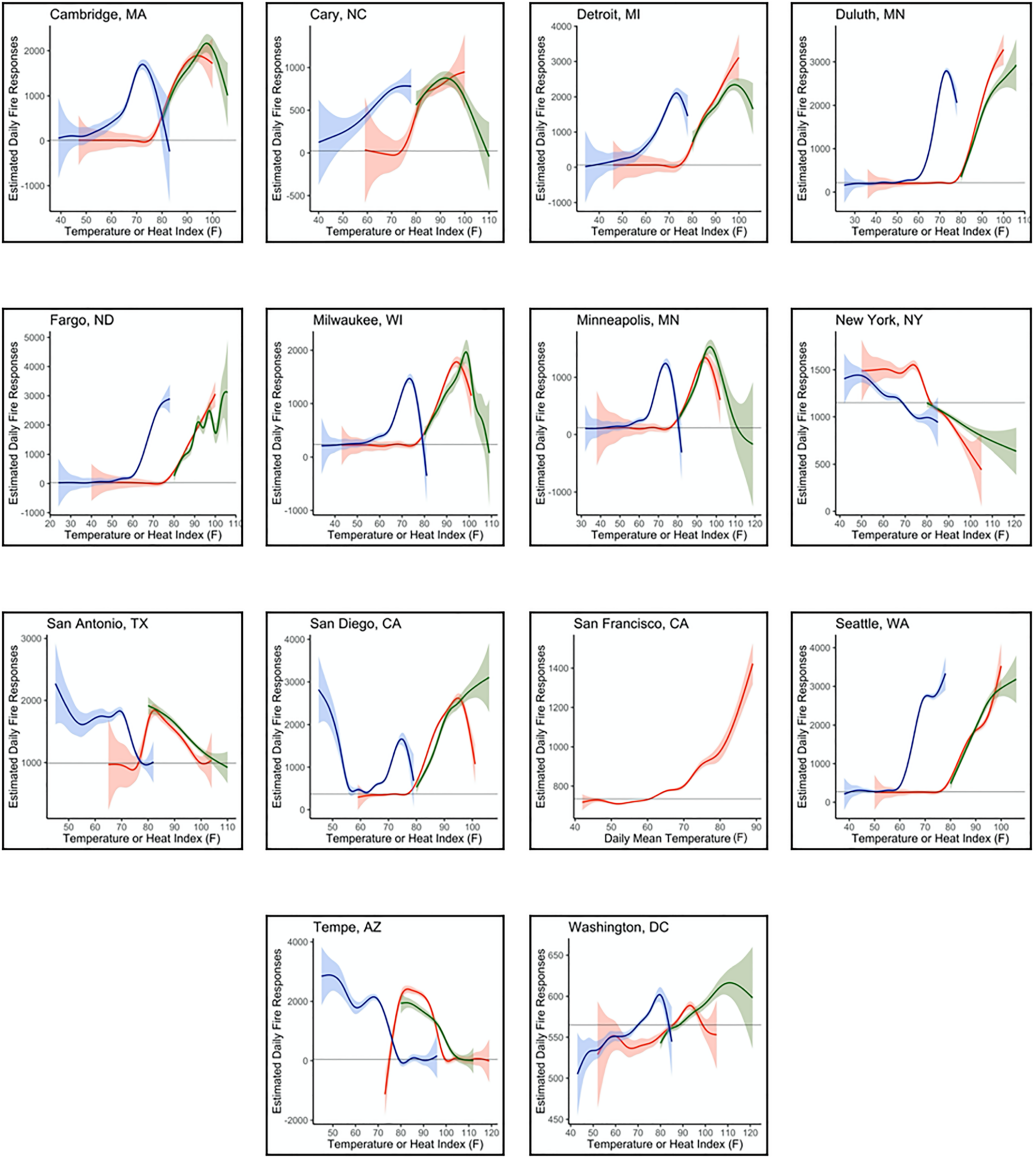
Estimated number of fire calls or incidents per unit temperature (°F) or heat index (°F) during the warm season, where red indicates daily maximum temperature, green indicates daily maximum heat index, and blue indicates daily minimum temperature during the preceding day, the mean number of fire calls/incidents for each city is shown by in black, and 95% confidence intervals are shaded around each respective line.

Similar to police incidents, the estimated effects of T_MAX_, HI_MAX_, and T_MIN_ on fire department incidents were nonlinear across all cities (Figure 3). Additionally, there were patterns in the TS analyses of fire department incidents between climate zones but not necessarily by city size, data type, or geographic region. For the cities in the *Very Cold* climate zone (Fargo, ND, and Duluth, MN), it appears that the estimated number of daily fire department incidents, on average, increased with all three temperature metrics analyzed. In the *Cold* cities (Cambridge, MA, Detroit, MI, Minneapolis, MN, and Milwaukee, WI) though, the curves were more inverse U‐shaped. These cities each demonstrated peaks in the estimated number of daily incidents with T_MAX_ or HI_MAX_ between 90°F and 100°F and T_MIN_ around 70°F. For Cambridge, MA, and Milwaukee, WI, the estimated number of incidents was greater when considering relative humidity and temperature (via HI_MAX_) than just temperature (T_MAX_). Trends were less clear across cities for the *Hot‐Humid* (San Antonio, TX), *Mixed‐Humid* (Washington, DC, New York, NY, and Cary, NC), and *Hot‐Dry* (San Diego, CA, and Tempe, AZ) cities.

The continuous and nonlinear values of T_MAX_ and HI_MAX_ were each significant predictors in warm‐season TS models for fire incidents in all cities except Tempe, AZ. During the warm season, a 10°F increase in T_MAX_—from 80°F to 90°F—resulted in increases in the expected number of daily fire department incidents in nearly all cities, ranging from 2.52% in San Antonio, TX, to 7.98% in Seattle, WA, to 11.4% in Detroit, MI. For a 10°F increase in the preceding day's T_MIN_ from 60°F to 70°F, the increase in the expected number of incidents was slightly reduced but still upward of approximately 6% in Cambridge, MA, and Duluth, MN.

## 4 Discussion

This study offers a comparative assessment of the impacts of heat on the frequency of police and fire department services in 23 U.S. cities during the warm season. Overall, there is an increased demand in urban fire and police services as daily maximum temperatures rise from ~80°F to over 100°F across the United States, regardless of city population size. These findings may be useful in informing climate change adaptation strategies, municipal budget, and personnel planning for emergency agencies to ensure resilience as periods of extreme heat increase in frequency, severity, and duration.

The RR of police department incidents on days where T_MAX_ was at or above the 95th percentile significantly increased from 3% (95% CI: 0.3%, 6.3%) to 57% (95% CI: 24.5%, 89.7%), compared to days below this threshold. The RR of fire department incidents increased from 6% (95% CI: 3.0%, 8.6%) to 18% (95% CI: 15.2%, 21.6%) at this temperature threshold. For most cities, the RR results remained consistent across temperature metrics and consecutive days of extreme heat. A 10°F increase in daily T_MAX_ from 80–90 °F resulted in a 3% increase in the expected number of daily police incidents in Cambridge, MA, and Hartford, CT, after adjusting for other predictors, as well as an 11% increase in the expected number of daily fire department incidents in Detroit, MI.

Our findings demonstrate increases in both fire and police department incidents during hot days, further supporting the body of evidence that extreme heat events have agency‐wide impacts on emergency services. Future analyses ought to elucidate the specific types of incidents that may be increasing on extreme heat days. In many cities, there were increases in the daily number of police or fire department calls from ~80°F to ~100°F with sharp declines in daily calls beyond 100°F. We hypothesize that above 100°F, human behavior changes such that people are more likely to stay indoors and thus are avoiding risks that emergency services may be responding to. This is well supported by past research which has demonstrated both decreases in physical activity (Obradovich & Fowler, 2017), police officer productivity (Obradovich et al., 2018), and policing intensity (Heilmann & Kahn, 2019) on the hottest days. As cities plan for more days over 100°F, it will be important to understand if human behaviors will become more acclimated to extremely high temperatures for planning of emergency services. In the hottest cities that were analyzed, including Austin, TX, New Orleans, LA, San Diego, CA, and Tempe, AZ, the preceding day's T_MIN_ resulted in the greatest number of estimated average daily police incidents, increasing as minimum temperatures decreased, which was unique. While we are unsure of the exact cause of this, human behavior may be playing a role that is worth future exploration. However, it is important to note that the coolest minimum temperatures (50–60°F) are rarer occurrences in these cities in hotter climate zones, and thus, there are wider CIs around these estimates.

There is ample research on the impacts of heat on emergency medical services and ambulance calls (Bassil et al., 2011; Calkins et al., 2016), violent, aggressive, and impulsive behaviors and crime (Anderson, 1989; Coccia, 2017; Gockel et al., 2014; Heilmann & Kahn, 2019), fatal traffic accidents (Leard & Roth, 2015), cognitive function (Cedeño Laurent et al., 2018), sleep (Obradovich et al., 2017; Williams et al., 2019), and a myriad of health outcomes (Armstrong et al., 2019; Basu, 2009; Reidmiller et al., 2018). Each of these pathways could result in increases in crime, traffic accidents, medical emergencies, or other emergency situations that require police or fire department response. However, there is limited research on the influence of heat on police and fire department services. Most research on the association between weather and climate variables with fire department services is focused on two topics: the increasing frequency of wildfires due to climate change (Hoegh‐Guldberg et al., 2018) or increases in residential fires from heating equipment and holiday decorations during cold weather (American Red Cross, 2006; Chandler, 1982).

Recent evidence has shown that there are nonwildfire‐related impacts of extreme heat on local fire departments during the warm season (Williams et al., 2020). Fire departments respond to many emergency scenarios, including medical emergencies and traffic accidents. Nationally, only 4% of fire department calls are for fires, while 64.3% are for medical incidents (US FEMA, 2018). This aspect of emergency services and the impact of extreme heat on their demand have been previously underrepresented in the scientific literature and in climate adaptation planning. A review of the municipal climate action plans for these 23 cities yielded only four mentions of heat's potential impacts on emergency services and their personnel needs.

This research elucidates the lesser‐studied impacts of extreme heat on police and fire department calls. The estimates within this study are similar to those found in the limited previous research on the impacts of extreme heat on police and fire department services. For example, a national analysis of various societal governance metrics in the United States demonstrated that police‐initiated stops increased up to approximately 29°C (84.2°F) and then decreased beyond this temperature, despite increases in police‐related violations (e.g., violence and driver error) (Obradovich et al., 2018). In Boston, MA, researchers found a 2% and 10% increase in the RR of police and fire department dispatches, respectively, on days where T_MAX_ was at least 90°F (Williams et al., 2020). The researchers also found that there were 1.016 and 1.002 times the expected number of police and fire department dispatches when T_MAX_ increased from 80–90°F in Boston, MA. The magnitude of these estimated increases is similar to those found within this study. Interestingly, the estimated increase for police (1.034) and fire (1.039) department incidents in Cambridge, MA—located just across the Charles River from Boston, MA—were larger than the estimates for Boston, MA.

There are a few limitations of this analysis that are important to consider. It is well noted that this lack of standardized metrics for emergency response data (Neusteter et al., 2019) has served as a challenge for robust research, and one large limitation of this study was that the type of data varied across cities, as did the data collection process between cities. Some cities only had police or fire call data available, while others had only incident report counts or calls related specifically to crime. We did not find any evident patterns in the results based on the data type used, but it is important to note the underlying differences in the dispatch systems and resulting data sets available between cities. The results of those cities that only had police calls related to crime are likely underestimates of the associations found, since this is just one type of calls that police departments respond to, and again, there were not distinct patterns for those cities that reported all police calls or crime. However, we recognize that this will require additional research with more granular data from all cities. Additionally, two cities (Chicago, IL, and Detroit, MI) only had one warm season of data that was available for analysis, and these estimates may not be representative of patterns in other warm seasons or over longer time periods.

This study was based on daily counts of fire and police incidents, and as a result, we are lacking information on the distribution of incidents within cities and on the reason for the call. We also did not have information on those utilizing these emergency services, so we could not do any further analyses on age, sex, other modifiable factors, or the geographic distribution of these services. Additionally, the coprovision of services from multiple emergency agencies at one time (i.e., we do not know for any given call if both BPD and BEMS were dispatched) is unknown. However, if a call warranted multiple agencies to arrive, there are still personnel and financial costs to those services that should be considered to best understand the full impact of extreme heat on these services in order to most appropriate plan for the future. This information would allow for additional evaluation of the impact of heat on specific types of emergencies that would benefit from adaptation and response planning. However, we felt it was important to first evaluate the full scope of the impact within these emergency response agencies to inform future, detailed analysis on emergency call types.

Lastly, there may be nondifferential exposure misclassification as a result of using ambient airport temperature exposures to define temperature. Temperature variations within and across urban areas might be different than the temperatures recorded at urban weather stations, which are commonly located at airports. In more densely populated cities, the urban heat island may even further exacerbate high temperatures. Further, many people in the United States spend a large majority of their time indoors (Klepeis et al., 2001), with elderly individuals, who are particularly vulnerable to extreme heat, spending upward of 90% of their day indoors. In cities with older housing stocks and lower air conditioning penetration, like in the Northeast United States, temperatures may be higher indoors for some individuals than they are outdoors. All of these would result underestimates of temperature exposures and resulting heat stress faced by residents of these cities.

Despite these limitations, police and fire department incidents increased with high temperatures in all cities, across many climate zones. This study reports the impact of heat on local police and fire department response across the entire respective agency. It also furthers the scientific understanding of heat's impact on police departments beyond violent crime. This underscores that in addition to the direct health consequences of extreme heat, particularly on vulnerable populations, extreme heat impacts our society and its vital functions, making it imperative for both mitigation of and adaptation to high temperatures.

As an example of evidence‐based planning, these results might be useful in informing climate change adaptation solutions for emergency service agencies. The increased use of police and fire department services on hot days may result in significant financial and personnel burdens. With an estimated 240 million 911 calls each year in the United States, even statistically small increases in daily call volumes can result in the need for greater staffing of personnel and additional resources. The expenditures for local fire protection services have already increased 196% from since 1980 due increased responsibilities for medical calls, increased staffing, and the rising cost of benefits (Evarts & Stein, 2020). Recent economic burdens at the local and state level due to the COVID‐19 pandemic will place further strain on public entities, like emergency response agencies. Policymakers and stakeholders should ensure that the increased costs of additional services on hot days are considered in future budgets and personnel plans.

In addition to planning for increased personnel and financial resources, local emergency services departments should also work to ensure occupational safety to heat stress for first responders, which has been found to increase on hot days (Xiang et al., 2014). There are 1.12 million fire fighters (Evarts & Stein, 2020) and 701,000 full‐time sworn police officers (Hyland, 2018) in the United States. Heat stress is one of the most frequently experienced injuries in fire fighters and law enforcement officials (FEMA, 2019; Houser & Science and Technology Policy Institute (Rand Corporation), 2004; University of Illinois at Urbana‐Champaign, 2008; US CDC, 2018). Traffic police officers have been found to have significant changes in blood pressure and heart rate (An et al., 2020), as well as heat stress symptoms like fatigue, cramps, and dizziness (Rasdi et al., 2017). Studies have found that firefighters can experience heat stress up to 20 times per year (Kim et al., 2019) and that many U.S. first responders agencies experience cases of heat illness regularly, with 39% of fire departments and 18% of law enforcement departments having at least one case of heat illness within the previous year (Bach et al., 2018). Despite this, nearly a quarter of the surveyed agencies provide no cooling mitigation or recovery resources (Bach et al., 2018). Climate change adaptation and preparedness plans need to account for the increased need of a broad number of societal services and these attendant consequences, like heat stress. More resilient departments, innovative deployments of temperature‐monitoring sensors, administrative safety measures (e.g., increased frequency of breaks and increased hydration support), and awareness of increasing risks of heat illness will be necessary to protect these first responders.

## 5 Conclusions

To date, there has been limited research comparing the impacts of extreme heat on police and fire department services across the United States. Through an evaluation of daily police and fire department incidents across 23 cities in six climate zones, we found significantly higher RR of incidents on hot days compared to nonhot days in many U.S. cities, although the shape of the associations differed. These results remained consistent across temperature metrics and consecutive days of extreme heat. Cities in cooler climates, like those found in the northern Great Plains, Midwest, and Northeast, had stronger associations when adjusting for daily, seasonal, and long‐term trends. A +10°F in daily T_MAX_ from 80–90°F resulted in 1.03 times the expected number of daily police incidents in Cambridge, MA, and Hartford, CT, on average after adjusting for other predictors, as well as 1.11 times the expected number of daily fire department incidents in Detroit, MI. These findings are vital to informing climate change adaptation strategies, preparing budgets and personnel for emergency agencies to ensure resilience as periods of extreme heat increase in frequency, severity, and duration.

## Conflict of Interest

The authors declare no conflicts of interest relevant to this study.

## Supporting information

Supporting Information S1

## Data Availability

All meteorological and emergency service data are available online (https://doi.org/10.5281/zenodo.3878968).
