# Supplementary material for: The Influence of Extreme Heat on Police and Fire Department Services in 23 U.S. Cities
Source: Geohealth. 2020 Nov 6;4(11):e2020GH000282. doi: 10.1029/2020GH000282 (PMC7648134; doi:10.1029/2020GH000282)
Supplement: Supplementary file 1 — Supporting Information S1 [file GH2-4-e2020GH000282-s001.docx]

**The Influence of Extreme Heat on Police and Fire Department Services in 23 U.S. Cities**

A. A. Williams^1,2^, L. McDonogh-Wong^2^, and J. D. Spengler^1^

^1^Harvard T.H. Chan School of Public Health, Department of Environmental Health

^2^Harvard T.H. Chan School of Public Health, Center for Climate, Health, and the Global Environment

Corresponding author: Augusta A. Williams ([auw882@harvard.edu](mailto:auw882@harvard.edu))

**Supplemental Materials**

Table S1. The relative risk (RR) of police and fire department calls meeting specific daily maximum heat index (HI_MAX_) or daily minimum temperature during the preceding day (T_MIN_) thresholds compared to all other days, calculated during the warm season. Bold values indicate RR’s that are significant at p<0.05

| **Agency** | **City** | **Humid Day** | | | | | | | **Warm Night** | | | | | | |
| --- | --- | --- | --- | --- | --- | --- | --- | --- | --- | --- | --- | --- | --- | --- | --- |
|  |  | **HI_MAX_ ≥ 95th percentile** | | | **HI_MAX_ ≥ 97th percentile** | | **HI_MAX_ ≥ 99th percentile** | | **T_MIN_ ≥ 95th percentile** | | **T_MIN_ ≥ 97th percentile** | | **T_MIN_ ≥ 99th percentile** | |  |
|  |  | RR | 95% CI | RR | | 95% CI | RR | 95% CI | RR | 95% CI | RR | 95% CI | RR | 95% CI |  |
| **Police** | Atlanta, GA | **1.05** | **(1.005, 1.102)** | 1.05 | | (0.990, 1.110) | 1.08 | (0.982, 1.178) | 1.01 | (0.968, 1.052) | 1.02 | (0.967, 1.073) | 1.02 | (0.930, 1.110) |  |
|  | Austin, TX | 0.98 | (0.917, 1.037) | 1.03 | | (0.955, 1.105) | 1.05 | (0.946, 1.154) | 1.01 | (0.954, 1.066) | 1.01 | (0.954, 1.066) | 1.02 | (0.929, 1.111) |  |
|  | Baltimore, MD | 0.97 | (0.931, 1.001) | 0.98 | | (0.930, 1.030) | 0.95 | (0.865, 1.035) | 0.99 | (0.958, 1.022) | 0.97 | (0.929, 1.011) | 0.97 | (0.905, 1.035) |  |
|  | Cambridge, MA | **1.07** | **(1.011, 1.129)** | 1.06 | | (0.987, 1.133) | 1.12 | (0.989, 1.251) | 1.02 | (0.965, 1.075) | 0.99 | (0.922, 1.058) | 0.99 | (0.859, 1.121) |  |
|  | Chicago, IL | **1.65** | **(1.316, 1.983)** | **1.60** | | **(1.193, 2.007)** | 1.62 | (0.938, 2.302) | **1.57** | **(1.210, 1.930)** | **1.61** | **(1.169, 2.051)** | 1.47 | (0.753, 2.187) |  |
|  | Detroit, MI | **1.13** | **(1.019, 1.232)** | **1.15** | | **(1.025, 1.275)** | 1.13 | (0.915, 1.345) | 1.08 | (0.971, 1.189) | 1.08 | (0.971, 1.189) | 1.17 | (0.959, 1.381) |  |
|  | Durham, NC | 1.03 | (0.970, 1.082) | 1.04 | | (0.976, 1.104) | 0.97 | (0.862, 1.078) | 1.01 | (0.953, 1.067) | 1.01 | (0.953, 1.067) | 1.01 | (0.923, 1.097) |  |
|  | Hartford, CT | 0.99 | (0.916, 1.061) | 0.99 | | (0.901, 1.079) | 1.05 | (0.908, 1.192) | 1.02 | (0.947, 1.093) | 1.03 | (0.942, 1.118) | 1.01 | (0.865, 1.155) |  |
|  | Los Angeles, CA | **1.04** | **(1.008, 1.067)** | **1.04** | | **(1.007, 1.073)** | 1.01 | (0.952, 1.068) | **1.03** | **(1.003, 1.057)** | 1.03 | (0.996, 1.064) | 1.05 | (0.995, 1.105) |  |
|  | Minneapolis, MN | 0.99 | (0.962, 1.009) | 0.98 | | (0.951, 1.009) | 0.92 | (0.864, 0.976) | 0.99 | (0.968, 1.012) | 1.00 | (0.973, 1.027) | 0.98 | (0.928, 1.032) |  |
|  | New Orleans, LA | **1.06** | **(1.033, 1.080)** | 1.02 | | (0.988, 1.052) | **1.05** | **(1.007, 1.093)** | 1.01 | (0.988, 1.032) | **1.04** | **(1.013, 1.067)** | 1.00 | (0.952, 1.048) |  |
|  | New York, NY | 1.00 | (0.985, 1.019) | 1.00 | | (0.978, 1.022) | 1.00 | (0.961, 1.039) | 1.00 | (0.984, 1.016) | 1.00 | (0.977, 1.023) | 1.00 | (0.962, 1.038) |  |
|  | Philadelphia, PA | 0.98 | (0.949, 1.003) | 0.98 | | (0.946, 1.014) | 1.00 | (0.940, 1.060) | 0.99 | (0.967, 1.013) | 0.99 | (0.954, 1.026) | 0.97 | (0.906, 1.034) |  |
|  | San Diego, CA | 1.02 | (0.994, 1.039) | **1.04** | | **(1.013, 1.067)** | 1.03 | (0.986, 1.074) | **1.03** | **(1.008, 1.052)** | **1.03** | **(1.008, 1.052)** | **1.06** | **(1.018, 1.102)** |  |
|  | Seattle, WA | 1.00 | (0.963, 1.032) | 0.97 | | (0.925, 1.015) | 0.95 | (0.886, 1.014) | 1.00 | (0.970, 1.030) | 0.98 | (0.942, 1.018) | 0.99 | (0.916, 1.064) |  |
|  | Tempe, AZ | 0.94 | (0.801, 1.080) | 0.94 | | (0.801, 1.079) | 1.00 | (0.697, 1.303) | 0.88 | (0.729, 1.031) | 0.88 | (0.729, 1.031) | 0.97 | (0.664, 1.276) |  |
| **Fire** | Cambridge, MA | **1.16** | **(1.048, 1.272)** | **1.21** | | **(1.057, 1.363)** | **1.34** | **(1.131, 1.549)** | 1.10 | (0.989, 1.211) | **1.16** | **(1.025, 1.295)** | 1.08 | (0.868, 1.292) |  |
|  | Cary, NC | **1.11** | **(1.047, 1.173)** | **1.13** | | **(1.043, 1.217)** | 1.06 | (0.911, 1.209) | 0.99 | (0.912, 1.068) | 0.99 | (0.912, 1.068) | 1.06 | (0.941, 1.179) |  |
|  | Detroit, MI | **1.13** | **(1.015, 1.245)** | 1.13 | | (0.994, 1.266) | 1.17 | (0.962, 1.378) | **1.20** | **(1.089, 1.311)** | **1.20** | **(1.089, 1.311)** | 1.18 | (0.953, 1.407) |  |
|  | Duluth, MN | **1.14** | **(1.108, 1.172)** | **1.15** | | **(1.109, 1.191)** | **1.15** | **(1.077, 1.223)** | **1.07** | **(1.040, 1.100)** | **1.09** | **(1.049, 1.131)** | **1.11** | **(1.046, 1.174)** |  |
|  | Fargo, ND | **1.14** | **(1.078, 1.202)** | **1.09** | | **(1.009, 1.171)** | 1.06 | (0.944, 1.176) | **1.06** | **(1.003, 1.117)** | **1.09** | **(1.010, 1.170)** | 1.12 | (0.997, 1.243) |  |
|  | Milwaukee, WI | **1.14** | **(1.104, 1.176)** | **1.15** | | **(1.106, 1.194)** | **1.19** | **(1.120, 1.260)** | **1.09** | **(1.056, 1.124)** | **1.10** | **(1.060, 1.140)** | 1.04 | (0.961, 1.119) |  |
|  | Minneapolis, MN | **1.21** | **(1.178, 1.242)** | **1.25** | | **(1.211, 1.289)** | **1.34** | **(1.269, 1.411)** | **1.07** | **(1.037, 1.103)** | **1.07** | **(1.030, 1.110)** | **1.14** | **(1.066, 1.214)** |  |
|  | New York, NY | **1.15** | **(1.122, 1.178)** | **1.18** | | **(1.144, 1.216)** | **1.19** | **(1.139, 1.241)** | **1.10** | **(1.072, 1.128)** | **1.10** | **(1.067, 1.133)** | **1.14** | **(1.082, 1.198)** |  |
|  | San Antonio, TX | 1.01 | (0.984, 1.036) | 1.03 | | (0.996, 1.064) | 1.05 | (0.980, 1.120) | 0.99 | (0.963, 1.017) | 0.99 | (0.963, 1.017) | 1.01 | (0.955, 1.065) |  |
|  | San Diego, CA | **1.20** | **(1.172, 1.228)** | **1.20** | | **(1.168, 1.232)** | **1.25** | **(1.191, 1.309)** | **1.15** | **(1.127, 1.173)** | **1.19** | **(1.152, 1.228)** | **1.19** | **(1.134, 1.246)** |  |
|  | Seattle, WA | **1.18** | **(1.112, 1.248)** | **1.19** | | **(1.104, 1.276)** | **1.15** | **(1.027, 1.273)** | **1.11** | **(1.050, 1.170)** | **1.14** | **(1.065, 1.215)** | **1.17** | **(1.020, 1.320)** |  |
|  | Tempe, AZ | 1.04 | (0.975, 1.105) | **1.11** | | **(1.018, 1.202)** | **1.17** | **(1.040, 1.300)** | 1.00 | (0.923, 1.077) | 1.00 | (0.923, 1.077) | 1.08 | (0.934, 1.226) |  |
|  | Washington, DC | **1.10** | **(1.070, 1.130)** | **1.12** | | **(1.081, 1.159)** | **1.11** | **(1.045, 1.175)** | **1.06** | **(1.029, 1.091)** | **1.07** | **(1.032, 1.108)** | **1.08** | **(1.017, 1.143)** |  |

Table S2. The number of police and fire department calls meeting specific daily maximum temperature (T_MAX_), maximum heat index (HI_MAX_) or minimum temperature during the preceding day (T_MIN_) thresholds calculated during the warm season.

| **Agency** | **City** | **Hot Day** | | | **Humid Day** | | | **Warm Night** | | |  |  |  |  |  |  |
| --- | --- | --- | --- | --- | --- | --- | --- | --- | --- | --- | --- | --- | --- | --- | --- | --- |
|  |  | **T_MAX_ ≥ 95th percentile** | **T_MAX_ ≥ 97th percentile** | **T_MAX_ ≥ 99th percentile** | **HI_MAX_ ≥ 95th percentile** | **HI_MAX_ ≥ 97th percentile** | **HI_MAX_ ≥ 99th percentile** | **T_MIN_ ≥ 95th percentile** | **T_MIN_ ≥ 97th percentile** | **T_MIN_ ≥ 99th percentile** |  |  |  |  |  |  |
| **Police** | **Atlanta, GA** | 36 | 23 | 8 | 31 | 20 | 7 | 44 | 26 | 9 |  |  |  |  |  |  |
|  | Austin, TX | 36 | 19 | 8 | 37 | 22 | 11 | 42 | 42 | 15 |  |  |  |  |  |  |
|  | Baltimore, MD | 99 | 47 | 12 | 67 | 32 | 11 | 82 | 49 | 19 |  |  |  |  |  |  |
|  | Cambridge, MA | 80 | 57 | 20 | 82 | 52 | 15 | 102 | 66 | 17 |  |  |  |  |  |  |
|  | Chicago, IL | 10 | 5 | 2 | 9 | 6 | 2 | 8 | 5 | 2 |  |  |  |  |  |  |
|  | Detroit, MI | 16 | 12 | 4 | 17 | 12 | 4 | 17 | 17 | 4 |  |  |  |  |  |  |
|  | Durham, NC | 41 | 25 | 15 | 44 | 33 | 12 | 44 | 44 | 18 |  |  |  |  |  |  |
|  | Hartford, CT | 31 | 20 | 7 | 34 | 22 | 8 | 33 | 22 | 8 |  |  |  |  |  |  |
|  | Los Angeles, CA | 69 | 48 | 16 | 69 | 57 | 18 | 88 | 54 | 19 |  |  |  |  |  |  |
|  | Minneapolis, MN | 115 | 79 | 31 | 111 | 71 | 20 | 123 | 83 | 22 |  |  |  |  |  |  |
|  | New Orleans, LA | 91 | 55 | 20 | 78 | 43 | 23 | 98 | 61 | 19 |  |  |  |  |  |  |
|  | New York, NY | 120 | 69 | 28 | 128 | 75 | 22 | 138 | 64 | 24 |  |  |  |  |  |  |
|  | Philadelphia, PA | 111 | 73 | 33 | 125 | 80 | 24 | 177 | 68 | 22 |  |  |  |  |  |  |
|  | San Diego, CA | 53 | 32 | 12 | 47 | 32 | 12 | 51 | 51 | 13 |  |  |  |  |  |  |
|  | San Francisco, CA* | 106 | 65 | 29 | -- | -- | -- | -- | -- | -- | -- | -- | -- | -- | -- | -- |
|  | Seattle, WA | 87 | 53 | 20 | 86 | 51 | 25 | 119 | 72 | 18 |  |  |  |  |  |  |
|  | Tempe, AZ | 86 | 53 | 13 | 73 | 73 | 14 | 66 | 66 | 14 |  |  |  |  |  |  |
| **Fire** | Cambridge, MA | 40 | 20 | 8 | 38 | 19 | 9 | 41 | 26 | 11 |  |  |  |  |  |  |
|  | Cary, NC | 71 | 41 | 15 | 68 | 34 | 12 | 49 | 49 | 19 |  |  |  |  |  |  |
|  | Detroit, MI | 22 | 17 | 5 | 21 | 15 | 6 | 21 | 21 | 5 |  |  |  |  |  |  |
|  | Duluth, MN | 109 | 61 | 22 | 107 | 64 | 20 | 131 | 69 | 27 |  |  |  |  |  |  |
|  | Fargo, ND | 42 | 27 | 12 | 40 | 24 | 12 | 53 | 25 | 10 |  |  |  |  |  |  |
|  | Milwaukee, WI | 87 | 51 | 25 | 91 | 58 | 22 | 105 | 74 | 20 |  |  |  |  |  |  |
|  | Minneapolis, MN | 115 | 79 | 31 | 110 | 71 | 20 | 123 | 83 | 22 |  |  |  |  |  |  |
|  | New York, NY | 51 | 37 | 10 | 53 | 32 | 16 | 61 | 43 | 13 |  |  |  |  |  |  |
|  | San Antonio, TX | 85 | 38 | 10 | 74 | 39 | 9 | 67 | 67 | 15 |  |  |  |  |  |  |
|  | San Diego, CA | 114 | 80 | 22 | 109 | 81 | 23 | 168 | 60 | 27 |  |  |  |  |  |  |
|  | San Francisco, CA* | 136 | 65 | 29 | -- | -- | -- | -- | -- | -- |  |  |  |  |  |  |
|  | Seattle, WA | 69 | 47 | 16 | 70 | 43 | 21 | 99 | 60 | 14 |  |  |  |  |  |  |
|  | Tempe, AZ | 24 | 15 | 6 | 34 | 15 | 7 | 24 | 24 | 6 |  |  |  |  |  |  |
|  | Washington, DC | 122 | 56 | 20 | 98 | 56 | 20 | 96 | 62 | 22 |  |  |  |  |  |  |

*Note: Instead of hourly temperature observations, San Francisco only had daily mean temperature available for this study period, so was calculated using the 95th, 97th, and 99th percentile of daily mean temperature.

Table S3. The relative risk (RR) of police and fire department calls meeting specific daily maximum temperature (T_MAX_), maximum heat index (HI_MAX_) or minimum temperature during the preceding day (T_MIN_) thresholds over 2 consecutive days compared to all other days, calculated during the warm season. Bold values indicate RR’s that are significant at p<0.05.

| **Agency** | **City** | **Hot Days (2 consecutive)** | | | | | | | **Humid Days (2 consecutive)** | | | | | | **Warm Nights (2 consecutive)** | | | | | | |
| --- | --- | --- | --- | --- | --- | --- | --- | --- | --- | --- | --- | --- | --- | --- | --- | --- | --- | --- | --- | --- | --- |
|  |  | **T_MAX_ ≥ 95th percentile** | | **T_MAX_ ≥ 97th percentile** | | **T_MAX_ ≥ 99th percentile** | | **HI_MAX_ ≥ 95th percentile** | | | **HI_MAX_ ≥ 97th percentile** | | **HI_MAX_ ≥ 99th percentile** | | **T_MIN_ ≥ 95th percentile** | | **T_MIN_ ≥ 97th percentile** | | **T_MIN_ ≥ 99th percentile** | |  |
|  |  | RR | 95% CI | RR | 95% CI | RR | 95% CI | RR | | 95% CI | RR | 95% CI | RR | 95% CI | RR | 95% CI | RR | 95% CI | RR | 95% CI |  |
| **Police** | Atlanta, GA | **1.10** | **(1.033, 1.163)** | 1.07 | (0.975, 1.161) | 1.10 | (0.955, 1.252) | **1.09** | | **(1.012, 1.168)** | 1.05 | (0.950, 1.150) | 1.10 | (0.951, 1.249) | 0.98 | (0.911, 1.049) | 1.07 | (0.977, 1.163) | 1.10 | (0.952, 1.248) |  |
|  | Austin, TX | 0.96 | (0.886, 1.038) | 0.92 | (0.799, 1.045) | 0.90 | (0.717, 1.088) | 0.99 | | (0.900, 1.080) | 1.03 | (0.920, 1.140) | 1.03 | (0.856, 1.204) | 0.99 | (0.901, 1.079) | 0.92 | (0.797, 1.043) | 0.90 | (0.715, 1.085) |  |
|  | Baltimore, MD | 0.98 | (0.943, 1.018) | 0.96 | (0.889, 1.022) | 0.96 | (0.677, 1.240) | 0.93 | | (0.870, 0.990) | 0.93 | (0.840, 1.020) | 0.95 | (0.809, 1.091) | 0.99 | (0.944, 1.036) | 0.96 | (0.893, 1.027) | 0.96 | (0.679, 1.241) |  |
|  | Cambridge, MA | 1.07 | (0.975, 1.169) | 1.06 | (0.945, 1.169) | 0.96 | (0.685, 1.231) | 1.07 | | (0.975, 1.165) | 1.05 | (0.914, 1.186) | 0.95 | (0.675, 1.225) | 0.99 | (0.911, 1.069) | 1.06 | (0.948, 1.172) | 0.96 | (0.687, 1.233) |  |
|  | Chicago, IL | **1.56** | **(1.109, 2.007)** | 1.47 | (0.750, 2.184) | 1.51 | (0.521, 2.506) | **1.62** | | **(1.243, 1.997)** | 1.52 | (0.941, 2.099) | 1.51 | (0.517, 2.503) | **1.55** | **(1.101, 1.999)** | 1.47 | (0.753, 2.187) | 1.51 | (0.517, 2.503) |  |
|  | Detroit, MI | **1.23** | **(1.044, 1.412)** | **1.24** | **(1.030, 1.440)** | 1.18 | (0.757, 1.595) | **1.21** | | **(1.062, 1.358)** | **1.23** | **(1.046, 1.414)** | 1.25 | (0.844, 1.656) | 1.11 | (0.933, 1.287) | **1.24** | **(1.035, 1.445)** | 1.18 | (0.761, 1.599) |  |
|  | Durham, NC | 1.04 | (0.953, 1.120) | 0.96 | (0.822, 1.088) | 0.88 | (0.702, 1.052) | 1.02 | | (0.940, 1.100) | 1.10 | (0.998, 1.202) | 0.95 | (0.732, 1.168) | 1.05 | (0.974, 1.126) | 0.96 | (0.827, 1.093) | 0.88 | (0.705, 1.055) |  |
|  | Hartford, CT | 0.97 | (0.846, 1.089) | 0.95 | (0.795, 1.114) | 0.99 | (0.579, 1.402) | 1.01 | | (0.896, 1.124) | 0.97 | (0.822, 1.118) | 1.03 | (0.848, 1.212) | 0.97 | (0.861, 1.079) | 0.95 | (0.791, 1.109) | 0.99 | (0.578, 1.402) |  |
|  | Los Angeles, CA | 1.03 | (0.987, 1.066) | 1.01 | (0.965, 1.063) | 1.01 | (0.919, 1.092) | 1.03 | | (0.991, 1.069) | 1.02 | (0.974, 1.066) | 0.97 | (0.882, 1.058) | **1.04** | **(1.006, 1.074)** | 1.01 | (0.961, 1.059) | 1.01 | (0.923, 1.097) |  |
|  | Minneapolis, MN | 0.98 | (0.942, 1.021) | 0.97 | (0.920, 1.022) | 0.93 | (0.852, 1.008) | 0.96 | | (0.922, 0.998) | 0.96 | (0.909, 1.011) | 0.91 | (0.822, 0.998) | 0.99 | (0.955, 1.025) | 0.97 | (0.919, 1.021) | 0.93 | (0.852, 1.008) |  |
|  | New Orleans, LA | 1.02 | (0.983, 1.047) | 1.04 | (0.994, 1.082) | 1.02 | (0.951, 1.089) | **1.05** | | **(1.015, 1.085)** | 0.97 | (0.913, 1.027) | 0.96 | (0.854, 1.066) | 1.02 | (0.991, 1.049) | 1.04 | (0.996, 1.084) | 1.02 | (0.951, 1.089) |  |
|  | New York, NY | 1.00 | (0.977, 1.023) | 1.00 | (0.972, 1.035) | 1.01 | (0.962, 1.067) | 1.00 | | (0.975, 1.025) | 0.98 | (0.943, 1.017) | 1.00 | (0.935, 1.065) | 0.99 | (0.967, 1.013) | 1.00 | (0.968, 1.032) | 1.01 | (0.957, 1.063) |  |
|  | Philadelphia, PA | 0.98 | (0.937, 1.023) | 1.01 | (0.952, 1.068) | 1.04 | (0.96, 1.127) | 0.96 | | (0.917, 1.003) | 0.94 | (0.879, 1.001) | 1.00 | (0.896, 1.104) | 0.97 | (0.937, 1.003) | 1.01 | (0.952, 1.068) | 1.04 | (0.957, 1.123) |  |
|  | San Diego, CA | **1.03** | **(1.005, 1.058)** | **1.04** | **(1.009, 1.077)** | **1.11** | **(1.037, 1.182)** | 1.02 | | (0.990, 1.050) | 1.01 | (0.971, 1.049) | 0.97 | (0.880, 1.060) | **1.03** | **(1.006, 1.054)** | **1.04** | **(1.006, 1.074)** | **1.11** | **(1.037, 1.183)** |  |
|  | San Francisco, CA* | **1.05** | **(1.010, 1.085)** | 1.04 | (0.990, 1.083) | 1.00 | (0.921, 1.082) | -- | | -- | -- | -- | -- | -- | -- | -- | -- | -- | -- | -- |  |
|  | Seattle, WA | 0.98 | (0.935, 1.031) | **0.93** | **(0.862, 0.998)** | 0.97 | (0.826, 1.110) | 0.98 | | (0.932, 1.028) | 0.97 | (0.905, 1.035) | 0.92 | (0.787, 1.053) | 1.00 | (0.959, 1.041) | 0.93 | (0.862, 0.998) | 0.97 | (0.828, 1.112) |  |
|  | Tempe, AZ | 0.96 | (0.782, 1.134) | 1.08 | (0.868, 1.290) | 1.24 | (0.786, 1.692) | 1.01 | | (0.821, 1.199) | 1.01 | (0.821, 1.199) | 1.24 | (0.656, 1.824) | 0.98 | (0.770, 1.190) | 1.08 | (0.869, 1.291) | 1.24 | (0.787, 1.693) |  |
| **Fire** | Cambridge, MA | 0.99 | (0.786, 1.192) | 1.01 | (0.688, 1.332) | 1.11 | (0.619, 1.591) | 1.14 | | (0.943, 1.337) | 1.11 | (0.713, 1.507) | 1.11 | (0.624, 1.596) | 1.10 | (0.940, 1.260) | 1.01 | (0.688, 1.332) | 1.11 | (0.624, 1.596) |  |
|  | Cary, NC | **1.14** | **(1.054, 1.220)** | **1.19** | **(1.079, 1.303)** | **1.42** | **(1.222, 1.618)** | **1.12** | | **(1.032, 1.208)** | 1.13 | (0.985, 1.275) | 1.18 | (0.899, 1.461) | 0.95 | (0.838, 1.062) | **1.19** | **(1.078, 1.302)** | **1.42** | **(1.222, 1.618)** |  |
|  | Detroit, MI | **1.27** | **(1.071, 1.469)** | **1.27** | **(1.051, 1.488)** | 1.27 | (0.779, 1.753) | **1.19** | | **(1.021, 1.359)** | **1.27** | **(1.071, 1.469)** | **1.50** | **(1.185, 1.815)** | **1.21** | **(1.021, 1.399)** | **1.27** | **(1.052, 1.488)** | 1.27 | (0.783, 1.757) |  |
|  | Duluth, MN | **1.17** | **(1.115, 1.219)** | **1.16** | **(1.081, 1.230)** | **1.26** | **(1.120, 1.398)** | **1.18** | | **(1.126, 1.234)** | **1.23** | **(1.158, 1.302)** | **1.22** | **(1.091, 1.349)** | **1.06** | **(1.014, 1.106)** | **1.16** | **(1.085, 1.235)** | **1.26** | **(1.121, 1.399)** |  |
|  | Fargo, ND | **1.12** | **(1.009, 1.234)** | 1.11 | (0.953, 1.271) | 1.10 | (0.710, 1.491) | **1.22** | | **(1.113, 1.327)** | **1.20** | **(1.059, 1.341)** | 1.10 | (0.710, 1.490) | 1.02 | (0.921, 1.119) | 1.11 | (0.951, 1.269) | 1.10 | (0.710, 1.490) |  |
|  | Milwaukee, WI | **1.17** | **(1.109, 1.238)** | **1.15** | **(1.071, 1.228)** | **1.21** | **(1.102, 1.318)** | **1.16** | | **(1.101, 1.219)** | **1.20** | **(1.129, 1.271)** | **1.29** | **(1.171, 1.409)** | **1.09** | **(1.040, 1.140)** | **1.15** | **(1.071, 1.229)** | **1.21** | **(1.102, 1.318)** |  |
|  | Minneapolis, MN | **1.21** | **(1.156, 1.265)** | **1.28** | **(1.21, 1.346)** | **1.30** | **(1.200, 1.405)** | **1.25** | | **(1.199, 1.301)** | **1.31** | **(1.243, 1.377)** | **1.35** | **(1.238, 1.462)** | 1.05 | (0.998, 1.102) | **1.28** | **(1.212, 1.348)** | **1.30** | **(1.197, 1.403)** |  |
|  | New York, NY | **1.17** | **(1.131, 1.212)** | **1.17** | **(1.120, 1.223)** | **1.17** | **(1.053, 1.293)** | **1.16** | | **(1.116, 1.204)** | **1.22** | **(1.162, 1.278)** | **1.23** | **(1.148, 1.312)** | **1.09** | **(1.051, 1.129)** | **1.17** | **(1.118, 1.222)** | **1.17** | **(1.050, 1.290)** |  |
|  | San Antonio, TX | **1.03** | **(1.005, 1.063)** | 1.01 | (0.956, 1.060) | 1.09 | (0.991, 1.195) | **1.05** | | **(1.012, 1.088)** | **1.06** | **(1.002, 1.118)** | 0.96 | (0.743, 1.177) | 0.99 | (0.946, 1.034) | 1.01 | (0.958, 1.062) | 1.09 | (0.988, 1.192) |  |
|  | San Diego, CA | **1.20** | **(1.159, 1.232)** | **1.20** | **(1.158, 1.246)** | **1.24** | **(1.134, 1.349)** | **1.21** | | **(1.175, 1.245)** | **1.23** | **(1.189, 1.271)** | **1.26** | **(1.153, 1.367)** | **1.17** | **(1.142, 1.198)** | **1.20** | **(1.156, 1.244)** | **1.24** | **(1.133, 1.347)** |  |
|  | San Francisco, CA* | **1.22** | **(1.179, 1.260)** | **1.29** | **(1.233, 1.353)** | **1.41** | **(1.301, 1.523)** | -- | | -- | -- | -- | -- | -- | -- | -- | -- | -- | -- | -- |  |
|  | Seattle, WA | **1.13** | **(1.062, 1.210)** | **1.15** | **(1.053, 1.247)** | 1.07 | (0.891, 1.278) | **1.20** | | **(1.107, 1.293)** | **1.22** | **(1.094, 1.346)** | 1.08 | (0.745, 1.415) | **1.10** | **(1.015, 1.185)** | **1.25** | **(1.129, 1.371)** | 1.04 | (0.622, 1.458) |  |
|  | Tempe, AZ | 1.03 | (0.923, 1.128) | 1.00 | (0.857, 1.139) | 0.99 | (0.777, 1.207) | 1.00 | | (0.909, 1.091) | 1.08 | (0.920, 1.240) | 1.16 | (0.917, 1.403) | 1.02 | (0.917, 1.123) | 1.00 | (0.859, 1.141) | 0.99 | (0.775, 1.205) |  |
|  | Washington, DC | **1.07** | **(1.027, 1.104)** | 1.02 | (0.961, 1.080) | 1.07 | (0.959, 1.181) | **1.10** | | **(1.059, 1.141)** | **1.12** | **(1.057, 1.183)** | 1.08 | (0.969, 1.191) | **1.08** | **(1.039, 1.121)** | 1.02 | (0.961, 1.079) | 1.07 | (0.959, 1.181) |  |

*Note: Instead of hourly temperature observations, San Francisco only had daily mean temperature available for this study period, so was calculated using the 95th, 97th, and 99th percentile of daily mean temperature.
